# Comparison of frequency domain and time domain methods for the numerical simulation of contactless ultrasonic cavitation

**DOI:** 10.1016/j.ultsonch.2022.106138

**Published:** 2022-08-26

**Authors:** Christopher Beckwith, Georgi Djambazov, Koulis Pericleous, Catherine Tonry

**Affiliations:** Computational Science and Engineering Group, University of Greenwich, 30 Park Row, London SE10 9LS, UK

**Keywords:** Ultrasonic melt processing, Numerical modelling, Acoustic cavitation, Acoustic resonance

## Abstract

The use of a top-mounted electromagnetic induction coil has been demonstrated as a contactless alternative to traditional ultrasonic treatment (UST) techniques that use an immersed mechanical sonotrode for the treatment of metals in the liquid state. This method offers similar benefits to existing UST approaches, including degassing, grain refinement, and dispersion of nanoparticles, while also preventing contact contamination due to erosion of the sonotrode. Contactless treatment potentially extends UST to high temperature or reactive melts. Generally, the method relies on acoustic resonance to reach pressure levels suitable for inertial cavitation and as a result the active cavitation volume tends to lie deep in the melt rather than in the small volume surrounding the immersed sonotrode probe. Consequently, (i) with suitable tuning of the coil supply frequency for resonance, the treatment volume can be made arbitrarily large, (ii) the problem of shielding and pressure wave attenuation suffered by the immersed sonotrode is avoided. However, relying on acoustic resonance presents problems: (i) the emergence of bubbles alters the speed of sound, resonance is momentarily lost, and cavitation becomes intermittent, (ii) as sound waves travel through and reflect on all the materials surrounding the melt, the sound characteristics of the crucible and supporting structures need to be carefully considered. The physics of cavitation coupled with this intermittent behaviour poses a challenge to sonotrode modelling orthodoxy, a problem we are trying to address in this publication. Two alternative approaches will be discussed, one of which is in the time domain and one in the frequency domain, which couple the solution of a bubble dynamics solver with that of an acoustics solver, to give an accurate prediction of the acoustic pressure generated by the induction coil. The time domain solver uses a novel algorithm to improve simulation time, by detecting an imminent bubble collapse and prescribing its subsequent behaviour, rather than directly solving a region that would normally require extremely small time steps. This way, it is shown to predict intermittent cavitation. The frequency domain solver for the first time couples the nonlinear Helmholtz model used for studying cavitation, with a background source term for the contribution of Lorentz forces. It predicts comparable RMS pressures to the time domain solver, but not the intermittent behaviour due to the underlying harmonic assumption. As further validation, the frequency domain method is also used to compare the generated acoustic pressure with that of traditional UST using a mechanical sonotrode.

## Highlights

• Development of a novel algorithm for improving the computational efficiency of time domain acoustic cavitation simulation.
• Coupling of a frequency domain acoustic cavitation model to a background Electromagnetics solver for the simulation of contactless ultrasonic processing of liquid aluminium.
• Both methods achieve similar results, but acoustic pressures obtained with the frequency domain solver are slightly lower, potentially due to influence from higher frequency harmonics which only exist in the time dependent solver.
• Both methods show that a contactless AC induction coil can reach the Blake threshold for acoustic cavitation through resonance, but attenuation due to cavitating bubbles then prevents further resonance and results in intermittent cavitation.

## 1 Introduction

Ultrasonic treatment (UST) of metals in the liquid state prior to casting has been shown to improve the mechanical properties of the resulting solidified metal. In the case of aluminium alloys, beneficial effects from UST include the reduction of trapped hydrogen (degassing), grain refinement, or the dispersion of particle clusters in metal matrix composites [1]. These benefits are often attributed to the formation and explosive collapse of bubbles in the melt (cavitation) due to large pressure oscillations [2]. At the point of collapse, extreme temperatures [3] and pressures [4] occur. Traditionally, UST experiments induce sound waves using an immersed mechanical sonotrode operating at low ultrasonic frequencies (17–20 kHz). This technique induces strong pressure waves, which are nevertheless attenuated rapidly away from the sonotrode tip due to intense inertial cavitation and gas shielding. The main sources of attenuation arise from thermal losses, viscous friction, and losses due to acoustic radiation [5]. This small, localized region of intense cavitation is then extended in practice via the use of mechanical stirring, or through the design of specialized container vessels that allow for the melt to make multiple passes through it, so treating a larger melt volume [6].

A contactless method has been suggested which addresses some of these problems [7]. This process should improve cast cleanness, as it eliminates the contamination that occurs with the traditional method due to sonotrode erosion via the use of an induction coil that does not contact the melt. This should also reduce cost as the traditional method requires frequent and expensive sonotrode tip replacements. Additionally, high temperature (Ni, Fe, Cu) or reactive (Ti, Zr) melts which are not suitable for traditional UST can be similarly processed with the contactless method. However, a significant issue that the contactless method must overcome, is that the sinusoidal component of the Lorentz Force, which is the main source of acoustic waves in the melt, is significantly weaker than the mechanical force generated by the vibrating sonotrode. 1D simulations of the pressure induced by a coil carrying 2 kA at 10 kHz result in 20 kHz vibrations with an initial amplitude of 2814 Pa [8], while the immersed sonotrode can reach initial pressures of over 1 MPa, depending on the peak to peak displacement. The pressure required for consistent cavitation occurs at the *Blake Threshold*, given by Equation (1), where $S=\frac{2\sigma }{p_{0}R_{0}}$ is the Laplace tension, $p_{0}$ represents atmospheric pressure, $R_{0}$ the equilibrium bubble radius, and σ the surface tension.

(1) $$P_{\mathrm{blake}}=p_{0}\left[1+\sqrt{\frac{4}{27}\frac{S^{3}}{1+S}}\;\right]$$

As an example, for a hydrogen bubble with an ambient radius of 10 µm in liquid aluminium, with σ=0.87 N/m and $p_{0}=101325$ Pa, the Blake Threshold occurs at approximately 153 kPa. Since this is over 75 times the pressure contribution from the induction coil, acoustic resonance is required to approach the pressures needed for processing to take place. Acoustic waves are therefore initiated at low pressure and rapidly reach resonance as a function of crucible geometry, melt volume and supply frequency due to the linear behaviour of acoustic propagation, whilst hydrogen remains dissolved in the melt. As bubbles emerge and grow due to rectified diffusion at higher acoustic pressures [9] and then begin rapid expansion at the Blake threshold, the acoustic propagation gradually becomes highly nonlinear and maintaining resonance becomes problematic. As pressures diminish as a result, the remaining gas dissolves back into solution and the cycle repeats. An important characteristic of the process is therefore intermittent cavitation.

To address this complex problem, this work presents a novel Frequency Domain model of the coupled nonlinear acoustic field and the Lorentz force induced by the induction coil. Frequency domain models have become an increasingly common method of solving sound propagation problems in bubbly liquids, due to their relative computational efficiency over time domain methods and for this reason a brief historical review of its development follows. An early model [10] incorporated the effect of bubbles on the acoustic field by including a complex valued wavenumber in the Helmholtz equation, but that was only suitable for low pressure, linear oscillations. Louisnard [11] then extended this approach with a semi-nonlinear model, reformulating the complex part of the wavenumber by taking into account the energy dissipation of large amplitude bubble oscillations, but still using the linear approximation for the real part. The effect of compressibility was then included by [5], who used the Keller-Miksis equation and found that the attenuation to acoustic radiation is comparable to that due to thermal dissipation, and the Keller-Miksis equation was again used by [12], who derived a fully nonlinear expression for the wavenumber from the Caflisch equations [13], and showed that the attenuation and sound speed depend strongly on frequency and pressure. A similar approach was then used by Trujillo [14] but initially only validated against the linear model at low acoustic pressures. This was then extended to higher pressure amplitudes and the model validated for cavitating liquids [15]. This model has also been used by [16], [17] for large acoustic pressure amplitudes and the simulation of acoustic streaming, and coupled to a solver for the fluid flow and solidification of liquid aluminium alloys. Jin et al. [18] compared Finite Element simulations of the linear and nonlinear Helmholtz models in modelling sonoreactors, and concluded that the linear model significantly overpredicts the acoustic pressure magnitude, so the use of nonlinear models is needed for accurate simulations.

On the other hand, time-dependent methods also have distinct advantages. Unlike frequency domain methods, they are not restricted to just one harmonic frequency, and can also address transient effects that harmonic solutions neglect [11]. A significant amount of existing literature here uses the full Caflisch equations [13] to model the sound propagation in bubbly liquids, including cavitating air bubbles in water, and hydrogen bubbles in liquid aluminium [19]. Many of these previous simulations have been restricted to two dimensions due to the computational requirements of resolving bubble collapses in each computational cell. Work by Vanhille et al. [20], [21] provides an alternative approach based on a damped oscillator equation, which has been used for the simulation of three dimensional acoustic fields [22]. However, this might not be valid for very high driving pressure amplitudes which result in significant increases in bubble volume fraction [23]. This work presents a new model featuring an algorithm which aims to improve the stability and efficiency of running the full Caflisch equations, and the results are compared to frequency domain simulations. In addition, simulations of the contactless method are then compared to results obtained using a traditional mechanical sonotrode.

## 2 Sound generation from the contactless sonotrode

**Fig. 1:**
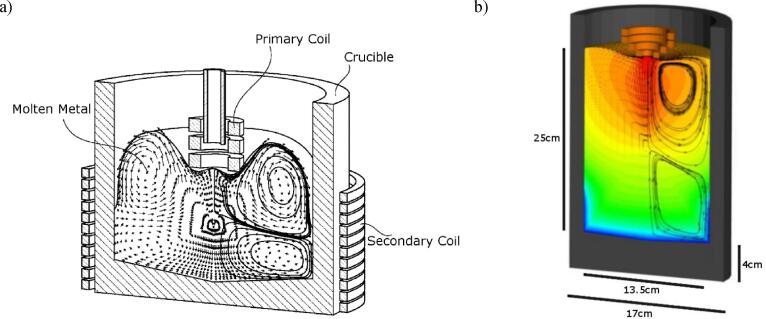
(a) The patented contactless sonotrode concept [24], (b) a schematic for the current experiments in a clay graphite cylindrical crucible.

In theory, any induction coil, including the one used for melting the metal charge can be used to generate vibrations in the melt. However, (a) the wave frequency needs to be around 20 kHz in agreement with current sonotrode practice, (b) to achieve maximum electromagnetic coupling between coil and melt, the distance between them must be minimised. Bearing in mind that opposite currents repel, this led to the idea of mounting the AC induction coil close to the melt free surface, as shown in the concept simulation in Fig. 1a [8]. The coil operates at a frequency between 6 and 10 kHz and with AC current up to 2000 A. During operation opposite currents are induced in the melt that result in a repulsive Lorentz force, which depresses the free surface of the melt preventing direct contact. A typical simulation reflecting the current experiments and the basis for the experimental data and numerical validation in this paper is presented in Fig. 1b, which shows the flow and temperature fields generated by the induction coil, as well as the resulting free surface depression.

The induced Lorentz force can be represented by F=J×B, where **J** and **B** are the current density and the magnetic flux density, respectively. With the AC coil assumed to operate at an angular frequency ω, the Lorentz force can then be represented by a time-averaged part $\overset{¯}{F}$, responsible for bulk mixing, and a time-dependent part $\tilde{F}$ which is the primary source of vibration. Considering the 1D case for clarity, this can be expressed analytically by Equations (2), (3) [25].

(2) $$\overset{¯}{F}=\frac{1}{2}\left(J_{r}B_{r}+J_{i}B_{i}\right)=\frac{1}{2\mu \delta }B_{0}^{2}e^{-\frac{2x}{\delta }}$$

(3) $$\tilde{F}=\frac{1}{2\mu \delta }B_{0}^{2}e^{-\frac{2x}{\delta }}\sqrt{2}\cos\left(2\omega t-\frac{2x}{\delta }+\frac{\pi }{4}\right)$$

Where $\delta =\sqrt{\frac{2}{\mu \omega \sigma }}$ is the skin depth, while the r and i subscripts indicate real and imaginary parts. Equation (3) demonstrates some important behaviour. First, the oscillation of the induced force operates at a frequency of 2ω, twice that of the AC frequency. Second, the force decays rapidly within the skin layer δ under the free surface. For liquid aluminium at an electrical frequency of 10 kHz, surface magnetic field amplitude of 0.1 T, electrical conductivity 2e6 S/m and magnetic permeability 4πx10^-7^H/m, this results in a skin depth of around 3.35 mm. With these parameters, the time-dependent part of the Lorentz force generates acoustic waves in the liquid metal with a typical amplitude of 2–3 kPa which is insufficient for the onset of cavitation and therefore acoustic resonance conditions must be established. In this initial low pressure phase, dissolved hydrogen in the liquid remains in solution and does not influence the acoustic wave propagation. As pressure oscillations begin to grow due to resonance, eventually a threshold is reached where hydrogen bubbles begin to grow due to rectified diffusion [26]. These bubbles attenuate the sound field and change the effective speed of sound in the liquid, which in turn changes the resonant frequency of the system preventing the pressure from building further.

**Fig. 2:**
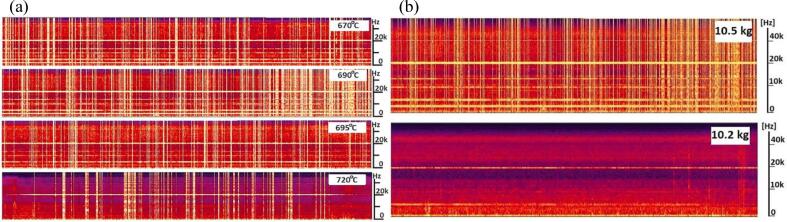
Spectrograms show the appearance of wideband noise bursts (vertical lines) [27] indicating the onset of cavitation. The horizontal lines, correspond to resonant peaks caused by the driving frequency in the top coil (∼20 kHz) and the melting coil (∼5 kHz). (a)Varying temperature between 670 °C and 720 °C shows maximum cavitation activity at 690 °C; by 720 °C most cavitation activity disappears. (b) Reducing the volume of metal from 10.5 Kg to 10.2 Kg, has a drastic effect on cavitation, with cavitation disappearing. Figures Dybalska et al..

Physical experiments using the immersed top coil clearly show evidence of this intermittent cavitation behaviour, indicated by the presence of broadband noise recorded sound spectra. Typical emitted sound spectrograms obtained in crucible experiments have been given in previous work [7], [28], [29], with example spectrograms presented in Fig. 2 for completeness. For the crucible described in Fig. 1b, spectrograms show the strongest response around 18.86 kHz and 18.42 kHz [28]. Other details of the experimental setup have also been given in previous publications, e.g. [6]. These spectrograms show the existence of broadband emissions that cannot be resolved by the high frequency microphone used and correspond to cavitation signals emitted by cavitating bubbles. The intermittent behaviour of these signals is apparent, and their density is a good indicator of the strength of cavitation. As shown further in Fig. 2, temperature affects cavitation intensity in aluminium, since it is easier to release hydrogen from solution close to liquidus, so activity decays as melt temperature increases from 670 °C to 720 °C. Changing the melt volume by taking a 300 g sample from the crucible eliminates cavitation, again showing sensitivity to overall melt dimensions to resonance. Further evidence of cavitation can be deduced post solidification from test samples cast from treated metal showing grain refinement [17], [28], [30] as well as degassing.

reproduced from [28]

## 3 Modelling approach

Time domain models based on the solution of Caflisch equations have previously been used to model the ultrasonic treatment of liquid metal alloys [8], [19], [31] with acoustic pressure predictions that match well with experimental data. The present model accounts for all acoustic frequencies which can be resolved effectively by the choice of the numerical scheme and grid size, including the sound emitted from higher frequency bubble oscillations. However, the solution of the full time-dependent system, becomes computationally expensive and impractical to use as very small timesteps - several orders smaller than the acoustic timestep, are needed to fully capture the behaviour of the bubble at collapse. On the other hand, frequency domain models based on a harmonic solution to the Caflisch equations have been used as a more efficient, but single frequency alternative [11], [14], [17], [32]. The nonlinear Helmholtz equation which results is then useful for running multiple simulations and performing a parameter estimation study or optimising model parameters including the sonotrode input power, temperature [32] operating frequency, or geometry [33] to maximize the intensity of cavitation.

A frequency domain study coupling a computational acoustics model with Maxwell’s equations to estimate the Lorentz forces acting on a skin layer under the melt free surface has previously been used by Tonry et al. [29] but was based on a linear Helmholtz model. This leads to a useful model describing the acoustic field within the melt and can be used to scan frequencies quickly to see which ones may result in resonance. However, a simple linear approach cannot predict the effect that inertial cavitation has on the sound field. This can lead to two main concerns. First, the amplitudes obtained by a linear acoustic model approach infinity as the driving frequency approaches the resonating frequency, which cannot happen due to inertial cavitation damping the acoustic waves. Secondly, variations in the speed of sound due to gas compressibility are not taken into consideration, which could lead to incorrect prediction of cavitating zone positions. However, since the initial pressure contribution due to the coil is approximately 2–3 kPa, and the effect of bubbles is negligible below the rectified diffusion threshold (approximately 83 kPa in the case presented here), the linear solution can be used to predict the resonant frequency of the system as resonance must exist to reach the threshold pressure at all.

Recognising the limitations of the frequency domain approach, in the following section we discuss improvements to the time domain method which aim to improve its computational efficiency by introducing a new algorithm, named “flipRdot”, for bubble behaviour near collapse. Similarly, improvements to the frequency domain method are then presented which enable the coupling to an electromagnetics study for the contribution of Lorentz forces.

### 3.1 Time domain model

A novel, computationally efficient method is proposed for solving the tightly coupled equations of acoustic cavitation in the time domain. As the oscillations of the cavitating gas bubbles significantly alter the acoustic field in the liquid volume, the nonlinearity of this phenomenon must be taken into account to determine accurately the resonant frequencies that can lead to the strongest pressure amplitudes, beneficial for the liquid metal treatment via cavitation.

As the Mach number in liquids is always negligibly small, the equations describing the acoustic motion in primitive variables reduce to.

(4) $$\frac{1}{\rho c^{2}}\frac{\partial p}{\partial t}+\nabla \cdot u=F+\frac{\partial \beta }{\partial t};\frac{\partial \beta }{\partial t}=\frac{\partial }{\partial t}\left(\frac{4}{3}\pi R^{3}N\right)$$

(5) $$\rho \frac{\partial u}{\partial t}+\nabla p=0$$

with *p* – the local acoustic time-dependent pressure, ρand *c* – the density and speed of sound in the liquid, ***u*** – the acoustic velocity vector, *t* – time, F – a source of sound resulting from the Lorentz force, *β*– bubble volume fraction, *R* – the instantaneous radius of the local bubbles (assumed spherical) and *N* [m^−3^] – the local concentration of bubbles. The rate of change of *β* which represents the effect of the oscillating bubbles on the sound waves is the main feature of the Caflisch model [13]. The bubble spherical oscillation, described by $\dot{R}$, depends on the variable local sound pressure *p*. This mutual dependence of *p* and *R* makes the model *tightly* coupled. A number of ordinary differential equations (ODE) have been proposed and used to model this motion; one providing a good balance between accuracy and complexity is the Keller-Miksis equation (8). It accounts partially (to 1st order) for the liquid compressibility in its rapid radial motion in the vicinity of the bubble. In the fully incompressible limit (c→∞), the Keller-Miksis equation [34] reduces to the original Rayleigh-Plesset equation [35].

As shown previously, [19] the time domain acoustic cavitation method involves the direct numerical solution of the Caflisch equations (4, 5, 8–10) where the acoustic partial differential equations (PDE) are tightly coupled with the highly nonlinear bubble dynamics ODE and the equation of state for the bubble gas. Extremely high gradients of the bubble variables develop at bubble collapse. The adaptive time step methods within the ODE solvers attempt to reduce the time step [19] at collapse to values which are so tiny that (a) are impractical for 3-dimensional simulations in realistic geometries, and (b) cast doubt over the arithmetic accuracy of the computed results due to roundoff even with enhanced computer precision.

**Fig. 3:**
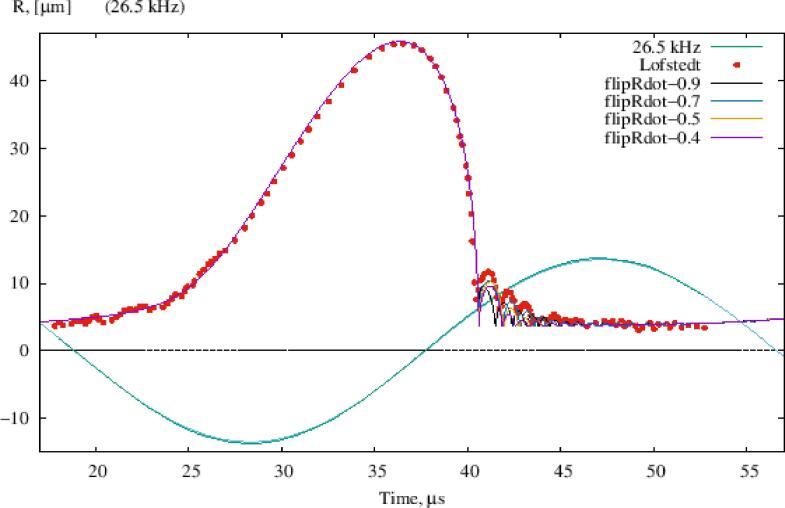
Response of air bubble with *R*_0_ = 4.5 μm in water to sinusoidal acoustic pressure with amplitude 135 kPa, *p*_0_ = 100 kPa, σ = 0.03 N/m, μ = 0.001 Pa.s, *c* = 1481 m/s [36].

Fig. 3 shows the typical bubble behaviour for one acoustic cycle compared to experimental data [36]. The driving acoustic pressure *p* has sufficiently high amplitude (above the Blake threshold for a given *R*_0_), so that explosive expansion of the gas bubble occurs, followed by rapid contraction, collapse, rebound on a time scale of a few nanoseconds and, possibly, several decaying secondary collapses (“after bounces”) [11]. The typical acoustic time step size for liquid metal in a laboratory-scale container would be between 50 and 100 ns (bearing in mind that the explicit time-stepping of the acoustic algorithm [19] must obey the CFL limit). The proposed new algorithm aims to avoid reducing this time-step beyond practical limits. To resolve the rebound, the ODE time step goes down to less than 0.005 ns which is prohibitively small when thousands of bubbles are simulated for hundreds of cycles.).

To address this problem, we consider an approximation to the bubble dynamics that provides stability gains while retaining an accurate representation of the physics and the effect of expanding and contracting bubbles on the ultrasound field. The proposed coupled algorithm ‘flipRdot’ for the Caflisch model proceeds as follows:

1. Solve the acoustic PDEs, mass continuity (4) and momentum (5) time-dependently, with time steps and mesh size appropriate for resolving the highest frequency in the desired range in the modelled geometry.
2. At each acoustic step, make a single, fully explicit ODE step with the Keller-Miksis equation (8) for all representative bubbles (located at the centres of the acoustic computational cells) calculating the *β* source term in (4).
3. Where, at the ODE step, an imminent bubble collapse is detected, invert the bubble motion by assigning a rebound bubble wall velocity and temporarily, for the current acoustic step, hold the bubble radius at a prescribed minimum value.
4. Repeat steps 1 to 3 until the end of the desired simulated time interval. (Usually, after the simulation, an FFT is carried out of acoustic pressure signals recorded at designated points in the solution domain. To achieve 10 Hz FFT resolution, the minimum simulated time interval is then 0.1 s.)

The parameters needed to control the proposed algorithm and their values used in the simulations for Fig. 3 are listed in Table 1.

**Table 1:** Parameters controlling the flipRdot algorithm.

Parameter	Value
Time step (for ODE and PDE, following CFL limit)	0.05 μs
Threshold minimum bubble radius coefficient	0.8
Threshold bubble wall velocity	−1 m/s
Bubble wall velocity inversion coefficient (variable parameter)	−0.9 to −0.4

These parameters are used as follows: Where the descending time-varying bubble radius *R* is less than 0.8 *R*_0_ and the bubble wall velocity $\dot{R}$ is less than −1 m/s, then bubble collapse is assumed imminent and the $\dot{R}$ reversal is done with the chosen coefficient in the current time step, while holding R = 0.8 *R*_0_. One can see in Fig. 3 that the value of the inversion coefficient only affects the ‘after bounces’. In the author’s experience, a value of −0.5 for this coefficient can be recommended; it means ¼ of the kinetic energy of the liquid associated with the bubble radial motion rebounds and ¾ is dissipated (into heat, light, sound and chemical energy).

This algorithm bypasses the singular collapse which occurs over several picoseconds, but might not fully capture the radiation and viscous losses which dominate during the collapse at very high pressures [5]. While this is a limitation of the model, this restriction can be overcome by fitting parameters in Table 1 to reproduce comparable rebound behaviour to the full ODE solution. In addition, since the top coil barely reaches Blake pressures, the error introduced in the acoustic pressure range of interest is small, and as the time dependent Caflisch solver is often implemented with the acoustics and bubble dynamics operating at different time scales [19] this region isn’t fully resolved by the acoustics code to begin with. The validation of this algorithm for the pressure range of interest then relies on its ability to correctly predict the bubble radii after collapse, and the ability to predict attenuation well enough to identify resonant frequencies comparable to that with more well established methods.

**Fig. 4:**
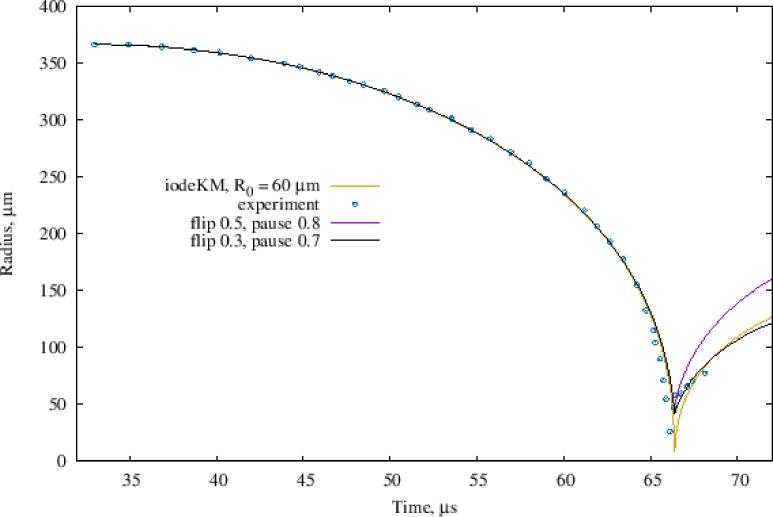
Collapse of a laser-induced bubble, initial *R* = 365 μm, in deionised water [37].

A validation example for a stronger bubble collapse [37] can be seen in Fig. 4. The yellow line (iodeKM) is the full numerical solution of the Keller-Miksis equation (8) with the Intel ODE solver. The ‘pause’ parameter is the minimum bubble radius relative to its equilibrium value. The ‘flip’ parameter is the negative bubble wall velocity inversion coefficient. Decreasing its value results in a weaker rebound, i.e. more energy is assumed to be dissipated by the collapse of the bubble. The bubble wall velocity is assumed as$\dot{R}$ = 0 at the beginning of the simulation; a threshold bubble radius (‘pause’) coefficient of 0.7 is seen to be more appropriate in this case. Away from the collapse and rebound region, the excellent agreement between model and experiment seen in Fig. 3 can be obtained with time steps up to 5 times higher than the value from Table 1; this means, especially for cases with liquid metal where *c* > 2000 m/s and with a required spatial resolution of 1 mm, the limiting factor is the acoustic CFL condition rather than the resolution of the bubble collapse which would be the case without the ‘flipRdot’ algorithm.

Overall, the parameters from Table 1 are subject to optimisation for classes of cases defined by the type of liquid and ranges of bubble size and driving ultrasound amplitude.

### 3.2 Frequency domain model

Starting from Equations (4), (5), and instead using the Lorentz force F in its volumetric form $F^{⋆}$ (units $N/m^{3}$) and treating it as a source of momentum rather than mass:

(6) $$\frac{1}{\rho c^{2}}\frac{\partial p}{\partial t}+\nabla \cdot u=\frac{\partial \beta }{\partial t};\frac{\partial \beta }{\partial t}=\frac{\partial }{\partial t}\left(\frac{4}{3}\pi R^{3}N\right)$$

(7) $$\rho \frac{\partial u}{\partial t}+\nabla p+F^{⋆}=0$$

Following the derivation procedure used by [14] in which the divergence of Equation (7) is subtracted from the first time derivative of Equation (6), it is possible to derive a nonlinear wave equation for the behaviour of the acoustic field including a background volumetric force. This is given in Equation (8).

(8) $$\rho \frac{\partial^{2}p}{\partial t^{2}}-\nabla^{2}p-{\nabla F}^{⋆}=\rho \frac{\partial^{2}\beta }{\partial t^{2}}$$

By dividing through by ρ and time averaging, following the standard derivation procedure of the nonlinear Helmholtz equation for cavitation problems [14] but now including this new source term, Equation (9) is obtained.

(9) $$\nabla \left(\frac{1}{\rho }\nabla P-\frac{F^{⋆}}{\rho }\right)+\frac{k_{m}^{2}}{\rho }P=0$$

The model in Equation (9) shares similarities to previous studies involving excitation from a sonotrode [11], [14], [16] but with an additional source term $F^{⋆}$. In addition, unlike existing approaches, the nonlinear Helmholtz equation used in this work differs by including terms which allow for variation in density. Density variations caused by the increase in bubble fraction are assumed to be small enough to be neglected, so the only density variations occur at material boundaries. The additional source term $F^{⋆}$ in this case is the harmonic amplitude of the volumetric Lorentz force, but in theory could be any volumetric force due to some background field. *P* represents the complex acoustic pressure field, and $k_{m}^{2}$ is a modified wave number given by Equation (10). The use of such a method does assume that the acoustic field can be approximated by only considering the main driving frequency, and it has been suggested that the pressure magnitude of other harmonics should be at least an order of magnitude lower than that of the main driving frequency [11].

(10) $$k_{m}^{2}={\left(\frac{\omega }{c}\right)}^{2}-\frac{A\left(P\right)}{\left|P\right|}-i\frac{B\left(P\right)}{\left|P\right|}$$

The dissipation functions A and B in Equation (10) take the same form as [14] and describe the change in speed of sound and attenuation due to the existence of inertially cavitating bubbles. To calculate these, the Keller-Miksis Equation (KME) [34] given in Equation (11) is used for the bubble dynamics simulation, as it accounts for the inclusion of liquid compressibility up to first order and contains additional acoustic radiation terms. Due to the Keller-Miksis equation only having compressibility terms accurate to first order, this model is only valid for Mach numbers $\dot{R}/c\ll 1$. In this work, this should be adequate as the acoustic pressures barely exceed the Blake threshold, but if compressibility is important, the KME should be replaced by a model with higher order terms, for example [38]. Temporal integration is calculated using the Tsitouras 5/4 Runge-Kutta algorithm (Tsit5) provided by the DifferentialEquations.jl library [39]. The flipRdot algorithm is not used this time, with the full solution including collapse modelled instead.

(11) $$\left(1-\frac{\dot{R}}{C}\right)R\ddot{R}+\frac{3}{2}{\dot{R}}^{2}\left(1-\frac{\dot{R}}{3c}\right)=\frac{1}{\rho_{l}}\left(1+\frac{\dot{R}}{c}+\frac{R}{c}\frac{d}{\mathrm{dt}}\right)\left[p_{l}-p\left(t\right)\right]$$

Then $p_{l}$ represents the liquid pressure at the liquid gas interface and is defined by Equation (12).

(12) $$p_{l}=p_{g}-\frac{2\sigma_{e}}{R}-\frac{4\mu \dot{R}}{R}$$

Where $\sigma_{e}$ is the surface tension, μ is the liquid viscosity, and $p_{g}$ is the pressure in the gas at the interface, which can be assumed to follow Equation (13), the adiabatic equation of state [40].

(13) $$p_{g}=p_{g0}{\left(\frac{R_{0}}{R}\right)}^{3\gamma }$$

**Fig. 5:**
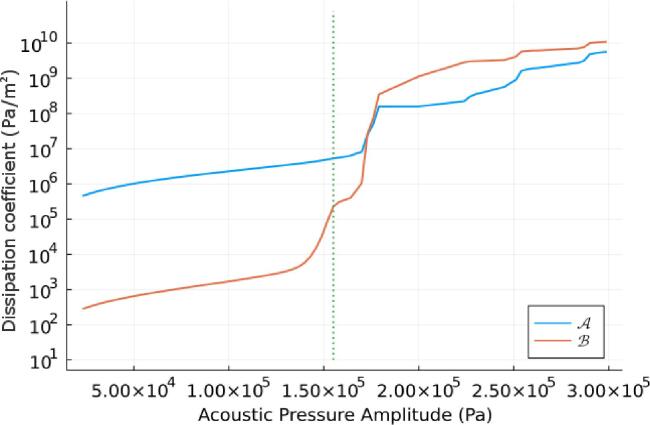
Change in nonlinear coefficients A and B with different driving acoustic pressure amplitudes. Large changes in the nonlinearity begin just before the Blake Threshold of 153370Pa for a 10 µm bubble. $p_{0}=1e5$ Pa, N = 1e8, and a frequency of 8.4kHz.

Where γ=1.4 is the polytropic exponent, and $p_{g0}$ the initial gas pressure in the bubble. The background pressure $p\left(t\right)=p_{0}\left(1-A\sin\left(\omega t\right)\right)$ accounts for the atmospheric pressure, and the sinusoidal acoustic pressure with dimensionless amplitude A, which is chosen to be 2.4, matching that of pressure readings from experiments in liquid Aluminium [41]. It should be noted that this equation of state assumes adiabatic conditions, and as a result, neglects the effect of thermal dissipation. Several authors have discussed how various physical effects contribute to dissipation, for example [11], [14], [42] and at higher acoustic pressures, acoustic radiation and viscous damping account for the vast majority of attenuation with values orders of magnitude higher than that of the attenuation due to thermal dissipation. However this is not true at low acoustic pressures where thermal dissipation can be significant, so this should be considered a limitation of the current model, with the potential to replace the equation of state with one that includes heat transfer, such as in Sojahrood et al. [42]. The variation of the nonlinear coefficients as the dimensionless amplitude increases is given in Fig. 5.

As the frequency domain solution assumes that the bubble oscillation has a harmonic component, it is important to run the single bubble model for more than one acoustic period so that simulation can converge to a harmonic solution. The number of cycles needed to reach a harmonic solution increases with frequency and driving amplitude [14], but at high pressure amplitudes a stationary solution cannot be achieved. In this case, the attenuation coefficients need to be interpolated. In the simulations in this paper, 500 cycles are chosen as the cut-off point at which if a harmonic solution has not been obtained, interpolation is used instead. The dispersion coefficients A and B can then be calculated using Equation (14) [14], which considers only the change in void faction over the last acoustic period.

(14) $$A=-\frac{\rho_{l}\omega^{2}}{\pi }\int_{0}^{2\pi }\frac{\partial \beta }{\partial \tau }\sin\tau d\tau ,B=-\frac{\rho_{l}\omega^{2}}{\pi }\int_{0}^{2\pi }\frac{\partial \beta }{\partial \tau }\cos\tau d\tau$$

Where $\beta =4/3\pi r^{3}N$ is the void fraction and N is the number of bubbles. In previous work [43], N was assumed to follow a smoothed stepwise function $W\left(\left|P\right|\right)$ centered on the Blake pressure, with an arbitrary smoothing distance in the range of 0.1 to 0.2 $P_{\mathrm{blake}}$. A downside to this approach is that it underestimates the effect of bubbles oscillating below this threshold, and as one of the main challenges to the top coil is the reliance on acoustic resonance to obtain cavitation this can lead to higher than realistic pressures, and an incorrect prediction of cavitation. In this work, we propose that a better approach is to choose this distance using the knowledge that bubbles will begin to grow once $P=P_{\mathrm{rd}}$, the rectified diffusion threshold, and as such the smoothing distance should be equal to $P_{\mathrm{blake}}-P_{\mathrm{rd}}$. A number of authors have investigated the process of rectified diffusion and have proposed equations for calculating $P_{\mathrm{rd}}$. In this work we will be following the work of Crum [9], and assuming that the driving frequency is far from the bubble resonant frequency, which is generally the case in metal processing. The resulting threshold is given by Equation (15), where $C_{i}$ is the concentration of hydrogen in the bulk fluid, $C_{0}$ is the saturation concentration, η is the polytropic coefficient.

(15) $$P_{\mathrm{rd}}^{2}=\frac{{\left(\rho R_{0}^{2}\omega_{0}^{2}\right)}^{2}\left[{\left(1-\omega^{2}/\omega_{0}^{2}\right)}^{2}\;+\;b^{2}{(\omega }^{2}/\omega_{0}^{2})\right]\left(1+2\sigma /R_{0}P_{\infty }-C_{i}/C_{0}\right)}{\left(3+4K\right)\left(C_{i}/C_{0}\right)-\left\{\left[\frac{3}{4}\left(\eta -1\right)\left(3\eta -4\right)\right]+\left(4-3\eta \right)K\right\}\left(1+2\sigma /R_{0}P_{\infty }\right)}$$

Another benefit to applying this smoothing is to improve the convergence of the method. While existing simulations using similar Non-Linear Helmholtz (NLH) based methods are concerned with the acoustic field generated by a sonotrode and are not concerned with resonance as the initial pressure amplitudes are high enough to trigger instantaneous cavitation, the contactless sonotrode relies on resonance to build up the pressure and then relies on intermittent cavitation to process the liquid. At near resonant frequencies, the NLH model suffers from bistability which prevents convergence, with the solution oscillating between the linear regime solution and the nonlinear solution.

The modified Helmholtz equation given in Equation (9), used for studying cavitation results in a system of heavily nonlinear equations, can take longer to converge when compared to the linear formulation [29], making full 3D frequency sweeps highly impractical. However, the linear model cannot be used to predict what pressures might be obtained in the presence of bubbles, or the size of the resulting active processing region which exists above the Blake threshold. This paper suggests an efficient method which provides the best of both approaches. The solution procedure follows 3 steps:

1. Perform an eigenfrequency study with a linear Helmholtz equation obtained by setting the number of bubbles *N* = 0, neglecting the effect of bubbles in the liquid. An assumption which should hold for the initial pressure build up before resonance.
2. Compute the induced harmonic Lorentz Force amplitude, by running a frequency domain Electromagnetics AC study at half the chosen eigenfrequency.
3. Once target resonant frequencies have been obtained, solve the nonlinear Helmholtz type model, given in Equation (9), which includes the effect of cavitating bubbles on the resulting field. As the resonant mode slightly shifts when including bubbles, it’s often required to perform a parametric study of frequencies close to the linear prediction to obtain resonance in the nonlinear model. The source term $F^{⋆}$ is set to the computed Lorentz Force amplitude computed in step 2, to get finite pressure amplitudes and the correct acoustic field.

## 4 Results

Both the time domain and frequency domain numerical models described in the previous chapter have been used to model the sound propagation in liquid aluminium with the geometry described in Section 2. The material properties used throughout both simulations are given in Table 2. In the time domain simulations, the Lorentz force was calculated from the 1D theory $\tilde{F}$ in Equation (3), while the frequency domain solution used the full Electromagnetics simulation, including the surrounding air and the copper coil. The effect of the clay graphite crucible was not considered in the Electromagnetics study as it is not electrically conducting but was considered in the acoustic study.

**Table 2:** Material properties for all materials used in the simulation.

Property	Aluminium	Clay Graphite	Air	Copper
Density ρ(kg/m^3^)	2375	1844	–	–
Speed of Sound c (m/s)	4560	1400	–	–
Relative permeability $\mu_{r}$	0.8	–	1	1
Electrical conductivity σ (S/m)	$2\times 10^{6}$	–	0	$5.99\times 10^{7}$
Relative permittivity ${∊}_{r}$	1	–	1	1

**Fig. 6:**
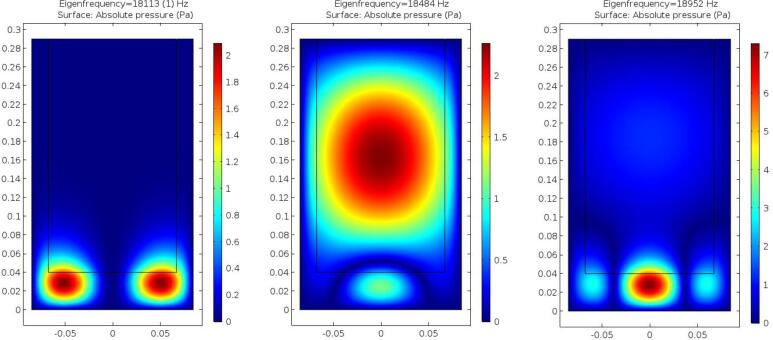
Eigenfrequency solutions for 3 chosen frequencies, demonstrates 2 modes which primarily act in the crucible wall, while the mode at 18484 Hz primarily acts on the liquid aluminium.

Three chosen eigenfrequencies for the cylindrical crucible obtained by using linear theory are shown in Fig. 6. These frequencies were chosen as they are close to the first peak recorded in the experimental data [28] and represent the closest frequencies obtainable by the coil. Of the chosen frequencies, only the 18484 Hz mode resonates the liquid aluminium, while the other modes primarily act on the crucible walls. To maximize cavitation in the liquid, the 18484 Hz mode will be the target mode in the following simulations.

**Fig. 7:**
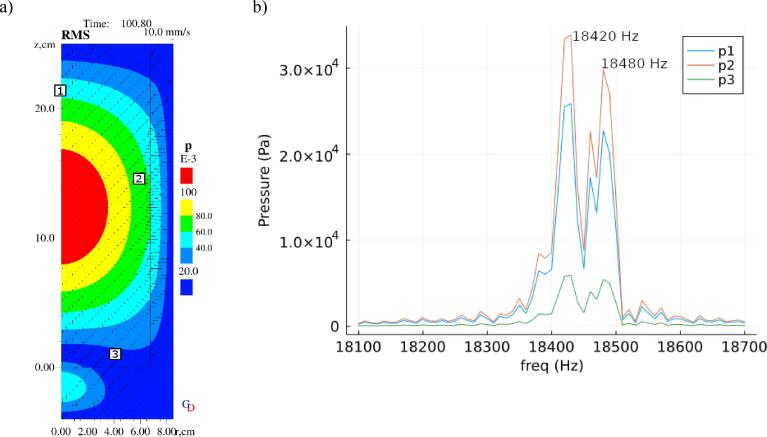
(a) RMS pressure distribution taken at T = 100.8 s with N = 1e8, (b) the frequency spectrum obtained by taking the FFT of the time series data at 3 node locations (r = 0, y = 21.5 cm), (r = 6 cm, y = 14.5 cm) and (r = 4 cm, y = 1 cm).

**Fig. 8:**
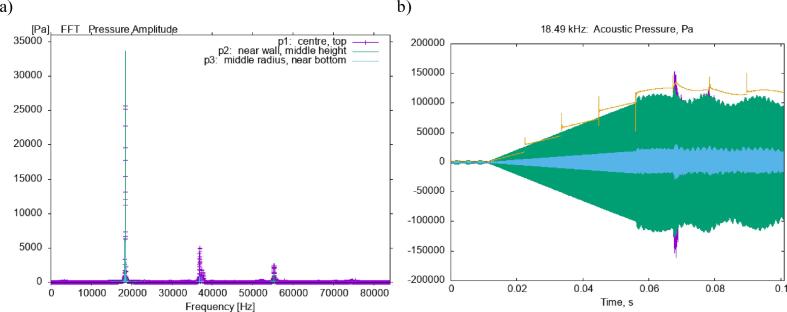
FFT spectrum from the time dependent study (a) shows the dominance of the driving, with a smaller contribution from higher frequency harmonics. Time series data (b) shows the initial linear pressure growth due to resonance, which then transitions into a region of intermittent cavitation.

The time domain model uses a fixed driving frequency of 18.49 kHz and N = 1e8. The choice of N is chosen such that the volume fraction of bubbles at the Blake threshold is comparable to previous simulations in liquid aluminium which used smaller bubbles [16]. The RMS pressure distribution is presented in Fig. 7a, and reaches a peak value of approximately 110 kPa, corresponding to a harmonic amplitude of approximately 155 kPa, just above the Blake threshold of 153.37 kPa. Taking the FFT at 3 locations in the domain shows the excitation of 2 main frequencies, at 18480 Hz and 18420 Hz, with an FFT resolution of 10 Hz. The FFT spectrum is provided in Fig. 8b. The pressure distribution closely matches the Eigenfrequency result in Fig. 6b, with the mode shifting 4 Hz due to the presence of bubbles.

The full FFT spectrum for the case is provided in Fig. 8a and shows the dominance of the driving frequency and its harmonics. Fig. 8b shows the time evolution of the acoustic pressure field, showing the initial linear growth due to resonance, with a transition to a nonlinear acoustic field around the rectified diffusion threshold of 83 kPa. The intermittent behaviour of the inertial cavitation can also be seen. As the pressure builds, more acoustic energy is lost due to attenuation, and the larger bubble oscillations also cause a shift in the speed of sound which results in the loss of resonance.

**Fig. 9:**
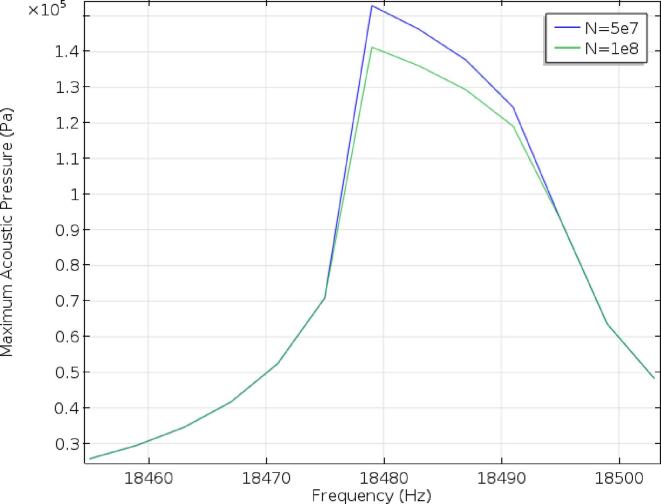
Maximum acoustic pressure obtained in the liquid aluminium for two bubble concentrations using the frequency domain method, with N = 5e7 and N = 1e8.

For the frequency domain study, the solution procedure described in Section 4 was carried out for frequencies close to the 18478 Hz predicted by the Eigenfrequency study. The peak pressures obtained in the liquid for frequencies ranging between 18450 Hz and 18510 Hz are given in Fig. 9. The highest acoustic pressure was achieved at 18479 Hz, close to the 18480 Hz predicted by the time dependent study and within the 10 Hz resolution provided by the FFT. Unlike the time domain model, the pressure required to obtain inertial cavitation could not be obtained with N = 1e8, but was obtained with N = 5e7, suggesting that the attenuation due to bubbles is strong enough just before the Blake threshold to prevent the transition from stable bubble oscillations to inertial cavitation. Another potential explanation for the slightly lower pressure could be that the higher frequency harmonics, which are neglected in the frequency domain study, contribute enough to make cavitation slightly easier to achieve numerically.

**Fig. 10:**
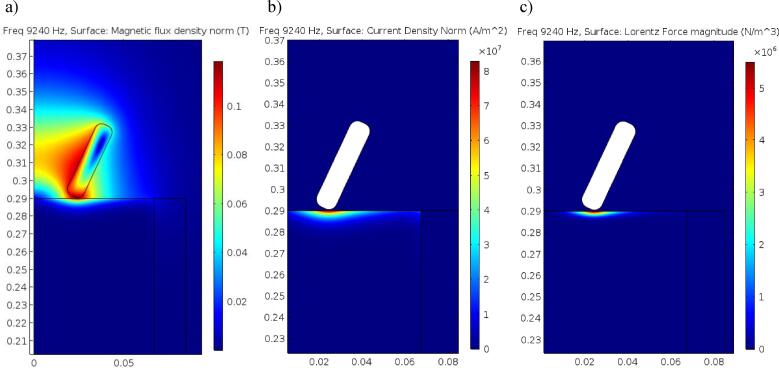
Magnetic flux density (a), Current density (b), and the Lorenz Force amplitude (c) in liquid Aluminium induced by the 3-turn copper induction coil operating with a current of 2000A, at 9240 Hz AC. The white region in (b) and (c) represents the 3 turn induction coil.

Fig. 10 shows the Magnetic flux density, Current density, and the time dependent component of the induced Lorentz Force for the 18479 Hz acoustic case where the strongest resonance was achieved, corresponding to 9240 Hz AC. The Magnetic Fields module in Comsol was used to solve for the induced Lorentz Force from the induction coil, and the amplitude of the induced Lorentz force is used by the acoustic solver as the source term $F^{⋆}$. The Magnetic flux density reached a peak magnitude of 0.11 T in the liquid aluminium skin layer, compared to the 0.1 T approximated by the 1D theory in Equation (3).

**Fig. 11:**
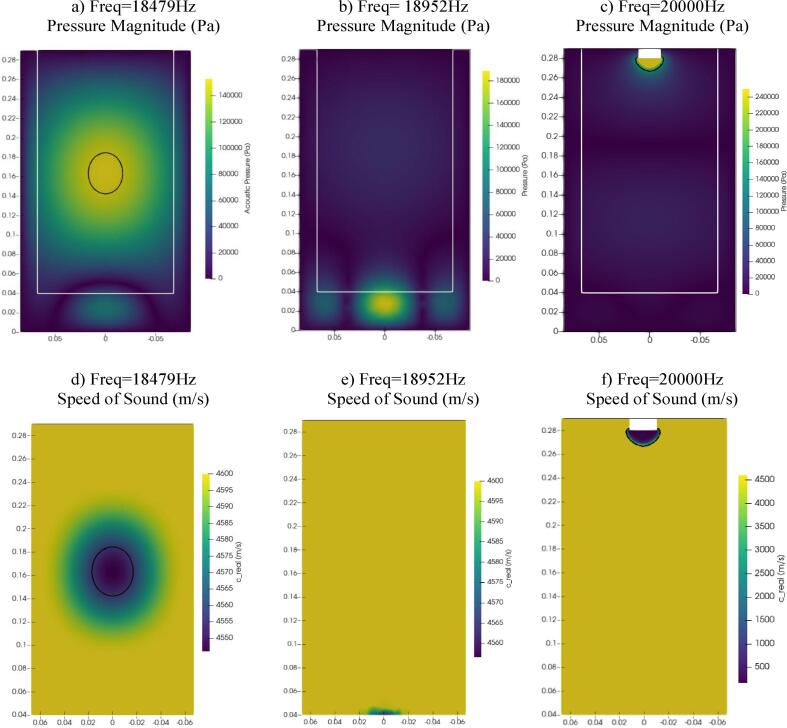
Acoustic pressure field predicted by the NLH model with N = 5e7 closely matches that of the linear solution for the top coil cases (a,b), but with pressure magnitude limited by the Blake threshold. Small localized cavities appear, which reduce the speed of sound (d,e), but the initial excitation from the Lorentz Force is too weak to significantly alter the field. For the traditional immersed sonotrode, the acoustic pressure field (c) differs significantly from the linear theory, and the cavity is localized to a thin area under the sonotrode. The black lines represent the region where $P>0.95P_{\mathrm{blake}}.$

The acoustic field calculated by the nonlinear Helmholtz solver is given in Fig. 11. The pressure distribution largely matches that of the linear acoustic theory, as seen in the Eigenfrequency results given in Fig. 6b and 6c. However, the pressure generated by the induction coil is not strong enough to overcome the rate of attenuation close to the Blake threshold, approximately 153.3 kPa for a bubble with an equilibrium radius of 10 µm in liquid aluminium. This agrees with the results obtained from the time dependent study, which showed the presence of intermittent cavitation. This contrasts with using a traditional vibrating sonotrode, which has an initial excitation several orders of magnitude greater, resulting in the nonlinear field differing from the linear theory significantly due to the constant cavitation under the sonotrode. This is shown in Fig. 11c, which presents the pressure distribution obtained by numerical simulation of a sonotrode with an 11 mm radius, operating at 20 kHz, and a peak-to-peak displacement amplitude of 24 µm. The pressure directly under sonotrode exceeds 250 kPa, with much stronger cavitation than what is obtained by the sonotrode, also causing higher bubble volume fractions and a greater shift in the speed of sound, down to a minimum of 280 m/s as can be seen in Fig. 11f.

## 5 Conclusions

A newly modified nonlinear Helmholtz model for cavitation has been demonstrated which accounts for variations in material properties and has been compared with a novel time dependent model using the Caflisch equations. Both models have accounted for the interaction of the sound wave with the clay graphite crucible, and the change in bubble density at low acoustic pressures. In addition, the new frequency domain model can easily be coupled with background sources of acoustic waves, such as Electromagnetically induced waves induced by Lorentz forces in the melt. The effect of heat transfer at the bubble interface was not included, as an adiabatic equation of state was used for simplicity, and both the time dependent and frequency domain models are limited only to low Mach numbers due to the use of the Keller Miksis equation.

Both models were applied to the case of a top-mounted induction coil as well as the more common immersed sonotrode technique, and the results show that while the induced pressure by the immersed sonotrode is much higher than that of the induction coil, altering the acoustic field significantly from the linear theory, it is still possible to get cavitation from the top coil as long as the bubble density is small enough for acoustic pressures below the Blake threshold, as the attenuation caused by oscillating bubbles can prevent resonance if the bubble volume fraction is sufficiently high. The number of bubbles is likely to depend on melt temperature in aluminium [32], but can also be influenced by degassing of hydrogen due to ultrasonic treatment [44], which has already been observed in experiments with the induction coil [28].

The results from both models showed very close agreement on the frequencies at which cavitation is likely to be found and matched well with the 18.42 kHz acoustic frequency at which cavitation was observed in experiments. However, the two methods differed slightly in the pressure magnitudes obtained, with the frequency domain study predicting slightly lower pressures. This is likely due to the contribution of higher frequency harmonics, which exist in the time domain study but are neglected in the frequency domain study. An advantage of using the frequency domain model is that it is possible to run simulations at multiple driving frequencies very efficiently, allowing for the much quicker discovery of optimum conditions for processing.

While sustained cavitation is unlikely when using the top coil due to the strong damping above the Blake threshold and the initial pressure contribution from the coil being significantly lower, intermittent cavitation is likely if resonance is reached, and this intermittency is shown with the time domain method. The resulting pressure field distribution from the top coil is then close to that predicted by the linear theory, but with peak pressures close to the Blake threshold. One significant difference between the two processing techniques is that while the immersed sonotrode focuses cavitation in a small region directly under the sonotrode, the top coil instead focuses cavitation at the antinode of a resonant mode, which can be deeper into the melt and more extensive. This coupled with electromagnetic stirring should enable the treatment of larger melt volumes.

## CRediT authorship contribution statement

**Christopher Beckwith:** Investigation, Methodology, Writing – original draft, Writing – review & editing. **Georgi Djambazov:** Investigation, Methodology, Writing – original draft, Writing – review & editing. **Koulis Pericleous:** Project administration, Supervision, Writing – original draft, Writing – review & editing. **Catherine Tonry:** Investigation, Validation.

## Declaration of Competing Interest

The authors declare that they have no known competing financial interests or personal relationships that could have appeared to influence the work reported in this paper.

## Data Availability

Data will be made available on request.
